# The efficiency of ^18^F-FDG-PET/CT in the assessment of tumor response to preoperative chemoradiation therapy for locally recurrent rectal cancer

**DOI:** 10.1186/s12885-021-08873-7

**Published:** 2021-10-21

**Authors:** Mamoru Uemura, Masataka Ikeda, Rio Handa, Katsuki Danno, Junichi Nishimura, Taishi Hata, Ichiro Takemasa, Tsunekazu Mizushima, Hirofumi Yamamoto, Mitsugu Sekimoto, Yuichiro Doki, Hidetoshi Eguchi

**Affiliations:** 1 Department of Gastroenterological Surgery, Graduate School of Medicine, Osaka University, 2-2 E2 Yamadaoka, Suita, Osaka, 565-0871 Japan; 2 Division of Lower GI Surgery, Department of Surgery, Hyogo College of Medicine, Hyogo, Japan; 3 Department of Surgery, Minoh City Hospital, Minoh, Japan; 4 Department of Surgery, Surgical Oncology and Science, Sapporo Medical University, Sapporo, Japan; 5 Department of Surgery, Kansai Medical University, Hirakata-City, Japan

**Keywords:** Locally recurrent rectal cancer (LRRC), PET-CT, Chemoradiation, Response assessment, Pathologic response

## Abstract

**Background:**

Locally recurrent rectal cancer (LRRC) remains a major problem after curative resection of primary rectal cancer. A noninvasive, prognostic biomarker with which to accurately evaluate disease status and assess the treatment response is critically needed to optimize treatment plans. This study assesses the effectiveness of PET/CT evaluation of preoperative chemoradiation therapy (CRT) in patients with LRRC.

**Methods:**

Since 2004, we have been performing preoperative CRT to improve local tumor control and survival. Between 2004 and 2013, 40 patients with LRRC underwent preoperative CRT (radiation: 50 Gy/25 fractions; chemotherapy: irinotecan plus UFT [tegafur and uracil]/leucovorin) and radical surgery, and underwent ^18^F-FDG-PET/CT before and 3 weeks after the completion of CRT. The maximum standardized uptake values (SUVmax) of the pre-CRT scan (Pre-SUV) and the post-CRT scan (Post-SUV) were measured. The predictive value of the ^18^F-FDG-PET and CT/MRI response assessments was evaluated.

**Results:**

The mean Pre-SUV was significantly higher than the Post-SUV (8.2 ± 6.1, vs. 3.8 ± 4.0; *P* < 0.0001). Following CRT, 17/40 patients (42.5%) were classified as responders according to the Mandard tumor regression grade (TRG1–2). The mean Post-SUV was significantly lower in responders than in nonresponders (2.0 ± 1.7 vs. 5.1 ± 3.9; *P* = 0.0038). Pathological response was not correlated with the response as evaluated by CT (*P* > 0.9999) or MRI (*P* > 0.9999). Multivariate regression analysis identified Post-SUV as an independent predictor of local re-recurrence-free survival (*P* = 0.0383) and for overall survival (*P* = 0.0195).

**Conclusions:**

PET/CT is useful in assessing tumor response to preoperative CRT for LRRC and predicting prognosis after surgery.

**Supplementary Information:**

The online version contains supplementary material available at 10.1186/s12885-021-08873-7.

## Background

Locally recurrent rectal cancer (LRRC) remains a major problem after curative resection of primary advanced rectal cancer [1]. The reported incidence of LRRC ranges between 5 and 30% after curative resection [2, 3]. Since 20 to 50% of these patients have local recurrence in the absence of distant metastasis, surgical intervention is one of the best curative treatment choices [3, 4]. Local control and long-term survival are possible for patients with isolated pelvic recurrence after extended radical operations, such as total pelvic exenteration. However, local re-recurrence and distant metastasis after resection of LRRC are relatively frequent [5]. We have been performing preoperative chemoradiation therapy (CRT) aiming to achieve local control and survival benefit [6]. Assessment of the tumor response is clinically important, but evaluation of the extent of LRRC by abdomino-pelvic computed tomography (CT) and pelvic magnetic resonance imaging (MRI) is sometimes difficult due to the main characteristics of LRRC, such as infiltrating growth, tissue scarring, and fibrosis [5].

^18^F-fluorodeoxyglucose positron emission tomography (^18^F-PDG-PET) is a powerful, noninvasive tool for imaging tumor metabolic activity [7] and particularly suitable to assessment of changes in tumor glucose metabolism after neoadjuvant treatment. The semiquantitative assessment of glucose metabolism by evaluation of the standardized uptake value (SUV) has been shown to have clinical relevance in evaluation of the response to CRT in several tumor types, including esophageal and advanced rectal cancer [8–10].

The objective of this study was to assess the effectiveness of ^18^F-FDG-PET/CT (PET-CT) evaluation of preoperative chemoradiation therapy (CRT) in patients with LRRC.

## Methods

### Patients

This retrospective cohort study was performed at Osaka University Hospital. Between 2004 and 2013, a total of 82 patients underwent resection for LRRC All patients had undergone a previous curative intent resection for primary rectal cancer without pre- or postoperative radiation therapy. Of these, 40 patients (26 male and 14 female) underwent preoperative CRT and radical surgery with pre- and postoperative PET-CT evaluation. Patients who had not received preoperative CRT for LRRC (*n* = 31) or patients who had not received pre- and postoperative PET-CT evaluation (*n* = 11) were excluded from this study. The extent of the recurrent tumor was evaluated by abdomino-pelvic CT, MRI, and colonoscopy. All patients underwent surgery between 3 and 6 weeks after completion of CRT. Resection was performed with curative intent on all patients. Patients were excluded from extensive pelvic surgery for posterior invasive LRRC when they exhibited distant metastasis or cancerous ascites. Sacral resections were performed only in the caudal regions (below S2) to preserve S1 sacral nerve function and to prevent walking disorders. Patients with apparent invasion to bone parenchyma of the side pelvic wall were also excluded from radical surgery. All study participants provided written informed consent. Re-recurrence was monitored with regular examinations at office visits and tumor marker assessments every 3 months for the first 3 years and every 6 months for the following at least 2 years, and CT scan every 6 months for at least 5 years.

### Pet/CT

Patients fasted at least 6 h before PET-CT scanning to minimize the blood insulin level and ensure standardized metabolism across patients. Blood glucose levels were determined just before FDG injection. All patients were normoglycemic (blood glucose < 150 mg/dL). Whole-body images were obtained 1 h after FDG injection (transmission source 68Ge-68Ga line source). Imaging was subsequently performed with a dedicated PET scanner (HEADTOME/SET 2400 W; Shimadzu Co, Kyoto, Japan). All patients received an PET/CT scan before CRT and another scan 3 weeks after completion of CRT. In a pilot phase of the study, 12 patients received an additional PET scan 2 weeks after the beginning of CRT as an interim assessment.

The maximum standardized uptake value (SUVmax) was calculated according to the following formula: PET count at most intense point ×calibration factor (MBq/kg)/injection dose (MBq)/body weight (kg). The SUVmax values of the pre-CRT scan (Pre-SUV), interim scan (Mid-SUV), and the post-CRT scan (Post-SUV) were measured. ∆SUV was defined by calculating the Pre-SUV–Post-SUV difference, and the percentage decrease between the Pre-SUV and the Post-SUV is presented as the decreasing rate (DR) = (∆SUV/Pre-SUV) × 100. Correlations between each of the SUV parameters (Pre-SUV, Post-SUV, ∆SUV, and DR) and pathologic tumor responses were analyzed.

### Preoperative chemo-radiotherapy

Preoperative radiation therapy of 50 Gy/25 fractions was delivered to the pelvis over 5 weeks (2 Gy/day for 5 days per week). Chemotherapy consisting of irinotecan (given biweekly at 30–60 mg/m^2^) plus UFT (tegafur and uracil)/leucovorin (given as a daily dose: 300 mg/m^2^/day and 75 mg/body/day, respectively) was administered concomitantly.

### Pathological assessment of response to preoperative CRT

Tumor response was assessed based on tumor viability and the extent of fibrosis and inflammation. Tumor regression grade (TRG), as described by Mandard et al. [11] in patients treated for esophageal cancer, was used to assess the pathologic tumor response after preoperative therapy. The TRG has been reported useful also for the assessment of tumor regression of rectal cancer [12]. TRG score induced by the neoadjuvant CRT was defined as follows: TRG1, complete regression with absence of residual cancer and fibrosis extending through the lesion; TRG2, presence of rare residual cancer cells scattered through fibrotic tissue; TRG3, increase in the number of residual cancer cells, with fibrosis predominant; TRG4, residual cancer outgrowing fibrosis; and TRG5, absence of regressive changes. We categorized responders as patients with TRG1 and TRG2 scores, while nonresponders had scores of TRG3 to TRG5. Resection was considered complete (R0) if a complete microscopic resection was confirmed regardless of the distance of surgical margin, and R1 resection was defined in cases where resection was macroscopically complete but microscopically incomplete.

### Response evaluation by CT and MRI

Tumor response after preoperative CRT was evaluated by CT and MRI after the completion of preoperative CRT, according to assessment using RECIST criteria [13]. MRI evaluation was made only based on RECIST criteria. Other evaluation methods such as the apparent diffusion coefficient, diffusion weighed signal, and the proportion of tumor compared to fibrosis [14], were not employed in this study. We defined responders as those patients obtaining a complete (CR) or partial (PR) response. CT and MRI evaluations were made by two radiologists independent of this study.

### Statistical analysis

Continuous data were expressed as median and range. Statistical analysis was performed using the χ^2^ test or Fisher’s exact test for categorical data. The paired t-test was employed to analyze SUV values. The Kaplan–Meier method was used to examine local re-recurrence-free survival, and the log-rank test was used to examine statistical significance. Prognostic factors were evaluated by univariate and multivariate analyses (Cox proportional hazard regression model). A value of *P* < 0.05 was considered significant. Statistical analysis was performed using JMP software (SAS Institute Inc., Cary, NC, USA).

## Results

### Patients and treatment for LRRC

A total of 26 males and 14 females were enrolled, with a median age of 68.5 years (36–81). All patients received radical resection with curative intent after pre-operative CRT. Among the 40 patients, 22 underwent total pelvic exenteration, while 10 patients underwent abdominoperineal resection, and 6 underwent low anterior resection**.** Sacral bone resection was concomitantly performed in 21 patients (52.5%) to secure a negative surgical margin. Resection was considered to be curative (R0 resection) in 36 patients and microscopically incomplete (R1) in 4 patients. According to Mandard’s criteria [11], 17 of the 40 patients were classified as responders (TRG1-TRG2) and 23 patients were classified as nonresponders (TRG3-TRG5). Clinical characteristics of the patients are described in Table 1. The median follow-up period after surgery was 53 (range 5.3–172) months.

**Table 1:** Patient Characteristics

Characteristic	Total (*n* = 40)
Sex	
Male	26
Female	14
Median age (years)	68.5 (36–81)
Median tumor size (mm)	33.5 (8.3–76.0)
Median CEA level pre-CRT (ng/ml)	9.0 (1.0–1022.0)
Median CEA level post-CRT (ng/ml)	3.0 (1.0–108.0)
Operation	
Tumorectomy*	2
Low anterior resection	6
Abdominoperineal resection	10
Total pelvic exenteration	22
Concomitant sacrectomy	
Done	21
Not done	19
Resection Status	
R0	36
R1	4
Tumor Regression Grade (TRG)	
TRG1–2	17
TRG3–4	23

*Tumor resection without adjacent organs

### Response assessment by PET-CT

Pre-SUVs ranged from 1.0 to 26.3 (8.2 ± 6.1, median 5.9). Post-SUVs ranged from 0.0 to 18.7 (3.81 ± 4.0, median 3.0), and Post-SUVs were found to be significantly lower than the pre-SUVs (*P* < 0.0001). The mean ⊿SUV was 4.4 ± 4.8 (range, − 1.3 ~ 22.5, median 2.8). The mean DR was 48.1 ± 30.3% (range, − 30.2 ~ 100, median 44.7%) (Table 2)**.**

**Table 2:** FDG uptake (all patients, n = 40)

	Mean ± SD	Median	Range
Pre-SUV	8.2 ± 6.1	5.9	1.0 to 26.3
Post-SUV	3.8 ± 4.0	3.0	0.0 to 18.7
∆SUV	4.4 ± 4.8	2.8	−1.3 to 22.5
DR(%)	48.1 ± 30.3	44.7	−30.2 to 100.0

Abbreviations: FDG,^18^F-fluorodeoxyglucose, SUV, standardized uptake value, Pre-SUV, SUVmax values on the initial scan, Post-SUV, SUVmax values on the post-CRT scan, ∆SUV, ∆SUV = Pre-SUV– Post-SUV, DR, decreasing rate, ∆SUV/Pre-SUV × 100(%), CRT, chemoradiation therapy

Twelve patients underwent PET-CT after the initial 2 weeks of CRT. In this pilot phase study, Post-SUVs were significantly lower than Pre-SUVs (*P* = 0.0442). However, Mid-SUVs were not significantly different from Pre-SUVs and Post-SUVs (*P* = 0.215 and 0.4068, respectively) (Supplementary Fig. 1)**.** Subsequent to the pilot phase, Mid-SUVs were no longer examined because this time point appeared to be too soon to assess the effect of CRT.

Post-SUVs in the responder (TRG1–2, *n* = 17) group were significantly lower than those in the nonresponder (TGR3–5, *n* = 23) group (2.0 ± 1.7 vs. 5.1 ± 3.9, *P* = 0.0038). DR (%) was significantly higher in the responder group than in the nonresponders (65.3 ± 32.3 vs. 35.4 ± 21.7, *P* = 0.0012). Pre-SUVs and ∆SUVs did not differ significantly between the responder and the nonresponder groups (*P* = 0.5103 and *P* = 0.2502, respectively) (Table 3).

**Table 3:** FDG-PET measurements and pathological classification

	Pathological responder TRG1–2 (*N* = 17)	Pathological nonresponder TRG3–5 (*N* = 23)	*P*-value
Pre-SUV	7.5 ± 5.2	8.8 ± 6.8	0.5103
Post-SUV	2.0 ± 1.7	5.1 ± 3.9	0.0038
∆SUV	5.4 ± 5.8	3.7 ± 3.8	0.2502
DR(%)	65.3 ± 32.3	35.4 ± 21.7	0.0012

Abbreviations: TRG the Mandard tumor regression grade, FDG-PET, ^18^F-fluorodeoxyglucose positron emission tomography, SUV, standardized uptake value, Pre-SUV, SUVmax values on the initial scan, Post-SUV, SUVmax values on the post-CRT scan, ∆SUV, ∆SUV = Pre-SUV– Post-SUV, DR, decreasing rate: DR = ∆SUV/ Pre SUV × 100(%), CRT, chemoradiation therapy

### Response assessment by CT and MRI

Table 4 shows the relationship between the CT/MRI response evaluation and pathological response grade. Most of the patients were classified in the nonresponder group by CT or MRI evaluation (26/40: 65.0% and 28/40: 70.0%, respectively), though almost half of these nonresponders were classified as responders using the pathological criteria (11/26: 42.3% and 11/28: 39.2%, respectively). There was no significant correlation between histological response classification and CT/MRI response classification (*P* > 0.9999 and *P* > 0.9999, respectively). Also of note is that CT or MRI was not useful in evaluating LRRC lesions in some cases (9/40: 22.5%, 3/40: 7.5%).

**Table 4:** CT and MRI evaluation of pathological grade

Response evaluation by CT				Response evaluation by MRI			
		Pathological responder (TRG 1–2) *N* = 17	Pathological nonresponder (TRG 3–5) *N* = 23			Pathological responder (TRG 1–2) N = 17	Pathological nonresponder (TRG 3–5) N = 23
CR	(CT responder)	0	0	CR	(MRI responder)	1	1
PR		2	3	PR		1	3
SD	(CT nonresponder)	11	15	SD	(MRI nonresponder)	11	17
PD		0	0	PD		0	0
ND		4	5	ND		1	2

Abbreviations: CR complete response, PR, partial response, SD, stable disease, PD, progressive disease, ND, not determined, RTG, tumor regression grade

### Local re-recurrence-free survival

**Fig. 1:**
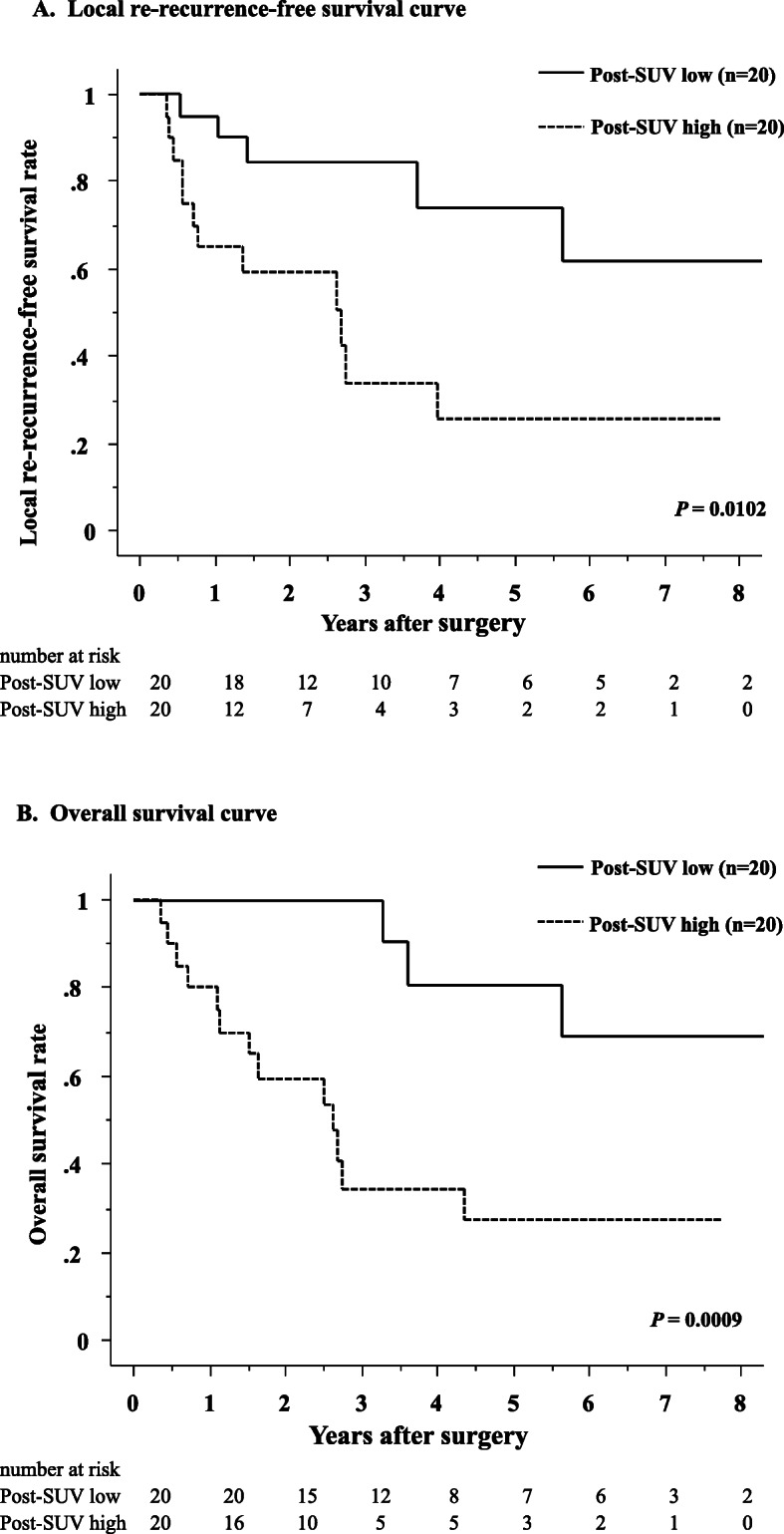
Kaplan-Meier local re-recurrence-free survival curve (A) and overall survival curve (B) for patients with LRRC, separated according to high and low Post-SUV

Patients’ age, sex, primary lesion-related factors, and locally recurrent lesion-related factors were analyzed using univariate analysis. The resection status and post-SUV were significant prognostic factors for local re-recurrence-free survival (*P* = 0.0299 and *P* = 0.0102, respectively). The median local re-recurrence-free time and 5-year local re-recurrence-free survival rate in the post-SUV low group was 9.4 years and 73.8%, respectively. In the post-SUV high group, it was 2.7 years and 25.3%, respectively (Fig. 1-A). When analyzed with these statistically significant parameters by univariate analysis, multivariate Cox regression analysis revealed that post-SUV was a significant prognostic factor (*P* = 0.0383) (Table 5).

**Table 5:** Univariate and multivariate analysis of prognostic factors for local re-recurrene-free survival

Variables	No. of patients	Univariate analysis	Multivariate analysis	
		*P*-value	Relative risk (CI)	*P*-value
**Age**				
≥ 62	20	0.2070		
< 62	20			
**Sex**				
Male	26	0.7584		
Female	14			
**Primary lesion-related factors**				
**Tumor differentiation**				
Well-differentiated	18	0.2617		
Others	17			
**Tumor depth**				
-MP	9	0.6736		
SS(A)-	31			
**TNM stage**				
I/II	21	0.4869		
III/IV	18			
**Venous invasion**				
(−)	13	0.3436		
(+)	21			
**Lymph node metastasis**				
(−)	26	0.4522		
(+)	13			
**Lymphatic invasion**				
(−)	7	0.5315		
(+)	27			
**Locally recurrent lesion-related factors**				
**Tumor size (maximal diameter)**				
< 34 mm	19	0.1067		
≥ 34 mm	19			
**CEA Pre-CRT**				
< 5 ng/ml	15	0.7853		
≥ 5 (ng/ml)	25			
**CEA Post-CRT**				
< 5 (ng/ml)	26	0.8175		
≥ 5 (ng/ml)	14			
**Resection status**				
R0	36	0.0299	0.433 (0.108–1.729)	0.2359
R1	4			
**Pathologic tumor response**				
Responders (TRG1–TRG2)	17	0.0601		
Nonresponders (TRG3–TRG5)	23			
**Pre-SUV**				
Low	20	0.5475		
High	20			
**Post-SUV**				
Low	20	0.0102	0.383 (0.104–0.940)	0.0383
High	20			
**∆SUV**				
Low	20	0.9160		
High	20			
**DR**				
Low	20	0.0488		
High	20			

Abbreviations: MP muscularis propri, SS, subserosa, A, adventitia, TNM, tumor node metastasis, TRG, the Mandard tumor regression grade, SUV, standardized uptake value, Pre-SUV, SUVmax values on the initial scan, Post-SUV, SUVmax values on the post-CRT scan, ∆SUV, ∆SUV = Pre-SUV– Post-SUV, DR, decreasing rate: DR = ∆SUV/ Pre SUV × 100(%), CRT, chemoradiation therapy

### Overall survival

In univariate analysis, resection status, pathologic tumor response, and Post-SUV were significantly associated with overall survival (*P* = 0.0035, *P* = 0.0411, and *P* = 0.0009, respectively). The median overall survival time and 5-year overall survival rate in the post-SUV low group was 8.4 years and 80.8%, respectively. In the post-SUV high group, it was 2.6 years and 27.2%, respectively (Fig. 1-B). Multivariate Cox regression analysis demonstrated that Post-SUV was a significant prognostic factor for overall survival (*P* = 0.0195) (Table 6).

**Table 6:** Univariate and multivariate analysis of prognostic factors for overall survival

Variables	No. of patients	Univariate analysis	Multivariate analysis	
		*P*-value	Relative risk(CI)	*P*-value
**Age**				
≥ 62	20	0.0893		
< 62	20			
**Sex**				
Male	26	0.5103		
Female	14			
**Primary lesion-related factors**				
**Tumor differentiation**				
Well-differentiated	18	0.5670		
Others	17			
**Tumor depth**				
-MP	9	0.7037		
SS(A)-	31			
**TNM stage**				
I/II	21	0.8033		
III/IV	18			
**Venous invasion**				
(−)	13	0.0560		
(+)	21			
**Lymph node metastasis**				
(−)	26	0.7551		
(+)	13			
**Lymphatic invasion**				
(−)	7	0.2828		
(+)	27			
**Locally recurrent lesion-related factors**				
**Tumor size (maximal diameter)**				
< 34 (mm)	19	0.6026		
≥ 34 (mm)	19			
**CEA (Pre-CRT)**				
< 5 (ng/ml)	15	0.9454		
≥ 5 (ng/ml)	25			
**CEA (Post-CRT)**				
< 5 (ng/ml)	26	0.6716		
≥ 5 (ng/ml)	14			
**Resection status**				
R0	36	0.0035	0.355 (0.084–1.501)	0.1590
R1	4			
**Pathologic tumor response**				
Responders (TRG1–TRG 2)	17	0.0411	0.576 (0.169–1.960)	0.3769
Nonresponders (TRG3–TRG 5)	23			
**Pre-SUV**				
Low	20	0.2012		
High	20			
**Post-SUV**				
Low	20	0.0009	0.203 (0.053–0.774)	0.0195
High	20			
**∆SUV**				
Low	20	0.6765		
High	20			
**DR**				
Low	20	0.1127		
High	20			

Abbreviations: MP muscularis propri, SS, subserosa, A, adventitia, TNM, tumor node metastasis, TRG, the Mandard tumor regression grade, SUV, standardized uptake value, Pre-SUV, SUVmax values on the initial scan, Post-SUV, SUVmax values on the post-CRT scan, ∆SUV, ∆SUV = Pre-SUV– Post-SUV, DR, decreasing rate: DR = ∆SUV/ Pre SUV × 100(%), CRT, chemoradiation therapy

## Discussion

A significant decrease in SUVmax was evident after CRT in this study. We observed that Post-SUV was especially useful in the assessment of LRRC survival, whereas CT and MRI less accurately reflected the pathological tumor response. Our data indicate that PET-CT is a useful imaging modality for the detection and evaluation of LRRC. PET-CT can distinguish cancer recurrence from postoperative scarring tissues or fibrosis [15–17] because it reports the metabolic activity of the region of interest. Consequently, PET-CT is ideally suited for imaging LRRC, considering the latter’s characteristic features of infiltrating growth, tissue scarring, and fibrosis [5].

In the current study, the Post-SUV was statistically lower in histopathologic responders than in nonresponders, suggesting that the effect of CRT can be predicted by the SUVmax. Moreover, the Post-SUV was a significant independent prognostic factor with respect to both local re-recurrence-free survival and overall LRRC survival (*P* = 0.0383 and *P* = 0.0195, respectively). According to several previous reports, a highly significant correlation was observed between a decreased FDG uptake rate after CRT and survival in patients with cancer of the esophagus [8, 17]. In these studies, multivariate regression analysis identified Post-SUV as an independent predictor of local re-recurrence-free survival and overall survival. These findings conclude that a low Post-SUV results from a decrease in the number of viable esophageal tumor cells, which may lead to better prognosis. Likewise, we find that low a Post-SUV in the treatment of LRRC is a sign of therapeutic response and indicative of better prognosis.

There is no current consensus regarding the best time to perform PET-CT to achieve optimal assessment of LRRC tumor response. Several reports have suggested the utility of early PET-CT assessment during treatment, due to the potential for modification of the subsequent treatment strategy [18–20]. However, our preliminary pilot study (*n* = 12) evaluating early PET-CT assessment during CRT (2 weeks after beginning CRT) failed to show positive results with respect to clinical utility in LRRC treatment. A possible explanation for this discrepancy is that LRRC cancer cells are exposed to a hypoxic environment in scar tissue and consequently might be more resistant to CRT than those in primary rectal cancers or esophageal cancers [5, 21]. We performed resection after 3 to 6 weeks following initiation of CRT, so we could not measure SUV at later time points. We decided to carry out the PET evaluation 3 weeks after completion of CRT, as previously described for primary cancers [22–24].

The major reason for the introduction of CRT into the course of treatment was to prevent local re-recurrence, which was previously found in 57.1% (12/21) of patients who received resection with curative intent and without preoperative CRT at our institution (detailed data not shown). After initiation of preoperative CRT for patients with LRRC, the local re-recurrence rate was still high (42.3%, 22/52, detailed data not shown). To improve the surgical outcome, it is important to be able to identify the patients with a high risk of local re-recurrence. We do not routinely perform adjuvant chemotherapy after the resection of LRRC, because patients’ conditions after surgery are widely variable, including postoperative complications such as pelvic abscess [7].

This study had some limitations. First, it is a single-center study. Second, the cohort was relatively small, though this is the first report discussing the usefulness of PET-CT for patients with LRRC.

## Conclusions

In conclusion, PET-CT is useful in the assessment of tumor response to preoperative CRT for patients with LRRC. Post-SUV and DR were significantly associated with a pathological treatment response. Post-SUV is especially valuable as an independent prognostic indicator for patients with LRRC.

## Supplementary Information

**Additional file 1: Supplementary Fig. 1.** Uptake of ^18^F-fluorodeoxyglucose measured in patients included in the pilot phase of the study (*n* = 12), in which Pre-SUV, Mid-SUV (2 weeks after initiation of CRT), and Post-SUV (3 weeks after completion of CRT) were determined. Mid-SUV was not significantly different from Pre-SUV and Post-SUV (*P* = 0.215 and *P* = 0.4068, respectively).

## Data Availability

The datasets generated and/or analyzed during the current study are not publicly available, due to the privacy of the enrolled subjects, but these may be requested from the corresponding author, upon reasonable request.

## Abbreviations

LRRC: Locally recurrent rectal cancer
CRT: chemoradiation therapy
UFT: Tegafur and uracil
SUVmax: The maximum standardized uptake values
TRG: The Mandard tumor regression grade
CT: Computed tomography
MRI: Magnetic resonance imaging
^18^F-PDG-PET: ^18^F-fluorodeoxyglucose positron emission tomography
SUV: The standardized uptake value
